# INTEROCEPTION ACROSS DOMAINS: ASSESSING THE ROLE OF AGE

**DOI:** 10.1093/geroni/igad104.2441

**Published:** 2023-12-21

**Authors:** Pietra Bruni

**Affiliations:** VA Connecticut / Yale School of Medicine, New Haven, Connecticut, United States

## Abstract

Interoception plays a prominent role in homeostasis, providing information about the internal state and condition of the human body. It is most typically measured using heartbeat tasks, though there are mounting criticisms against focusing exclusively on signals that originate from the cardiac domain. While prior research has shown that variability in interoceptive ability exists when examined in relationship to numerous health, biological, and psychiatric factors, the role age holds remains largely unexamined. The primary goal of this study was to assess interoception in two distinct domains, cardiac and respiratory, across a community sample (n = 40) of twenty younger (mean age = 24.9 years) and twenty older (mean age = 69.3 years) adults. Two laboratory tasks measured interoception: a Heartbeat Tracking Task and a Respiratory Discrimination Task. Three measures of interoception were extracted: Interoceptive accuracy (IAcc), Interoceptive awareness (IAwe), and Interoceptive sensibility (ISen). Results from this experiment indicated that IAcc and IAwe were not correlated across domains. Of note, a significant difference was found between the two estimates of IAcc, indicating that participants were more accurate at assessing interoception in the respiratory than in the cardiac domain. Furthermore, younger adults (M= 0.49, SD = 0.29) exhibited significantly higher IAcc than older adults (M= 0.67, SD = 0.23); (t(36) = -2.04, p < 0.02) in the cardiac domain. These results indicate dissociable outcomes for two methods of assessing interoception, as well as illustrate the potentially prominent role age may hold when examining variability in interoceptive ability across domains.

